# Cytotoxic Activity and Composition of Petroleum Ether Extract from* Magydaris tomentosa* (Desf.) W. D. J. Koch (Apiaceae)

**DOI:** 10.3390/molecules20011571

**Published:** 2015-01-16

**Authors:** Giuseppina Autore, Stefania Marzocco, Carmen Formisano, Maurizio Bruno, Sergio Rosselli, Mariem Ben Jemia, Felice Senatore

**Affiliations:** 1 Department of Pharmacy, University of Salerno, Via Giovanni Paolo II 132, Salerno 84084, Italy; E-Mail: marzocco@unisa.it; 2 Department of Pharmacy, University of Naples “Federico II”, Via Domenico Montesano, Napoli 49 80131, Italy; E-Mails: caformis@unina.it (C.F.); fesenato@unina.it (F.S.); 3 Department STEBICEF, University of Palermo, Viale delle Scienze, Parco d’Orleans II, Palermo 90128, Italy; E-Mails: maurizio.bruno@unipa.it (M.B.); sergio.rosselli@unipa.it (S.R.); 4 Laboratoire des Plantes Extremophiles—Biotechnologic Center Borj-CedriaTechnopark, B.P. 901, Hammam-Lif 2050, Tunisia; E-Mail: meriem_cbbc@yahoo.fr

**Keywords:** *Magydaris tomentosa*, coumarins, furanocoumarins, xanthotoxin, xanthotoxol, isopimpinellin, osthole, bergaptene, MCF-7

## Abstract

The petroleum ether extract of *Magydaris tomentosa* flowers (Desf.) W. D. J. Koch has been analyzed by GC-MS. It is mainly constituted by furanocoumarins such as xanthotoxin, xanthotoxol, isopimpinellin, and bergaptene. Other coumarins such as 7-methoxy-8-(2-formyl-2-methylpropyl) coumarin and osthole also occurred. The antiproliferative activity of *Magydaris tomentosa* flower extract has been evaluated* in vitro* on murine monocye/macrophages (J774A.1), human melanoma (A375) and human breast cancer (MCF-7) tumor cell lines, showing a major activity against the latter.

## 1. Introduction

*Magydaris tomentosa* (Desf.) W. D. J. Koch ex DC is a plant of Apiaceae family having the synonyms of *Magydaris pastinacea* (Lam.) Paol. It grows in few regions of Mediterranean area and in Sicily it has been found in some mountain places [1]. Like most plants belonging to the family Apiaceae, its aerial parts are very rich in several coumarins and furanocoumarins (psoralenes), as pointed out in our previous investigation on its acetonic extract [2]. Interestingly, it shows the peculiar characteristic of containing diterpenes with an irregular skeleton [3]. No investigations on the composition and activity of its petroleum ether extract have been previously reported.

## 2. Results and Discussion

*Magydaris tomentosa* attracted our attention because it quickly turned the skin of the hands persistently dark when touched. This phenomenon is probably due to low polarity compounds occurring in trichomes of the plant’s organs breaking under pressure. Therefore we decided to investigate the petroleum ether extract obtained by infusion of the flowering tops of *M. tomentosa*.

**Figure 1:**
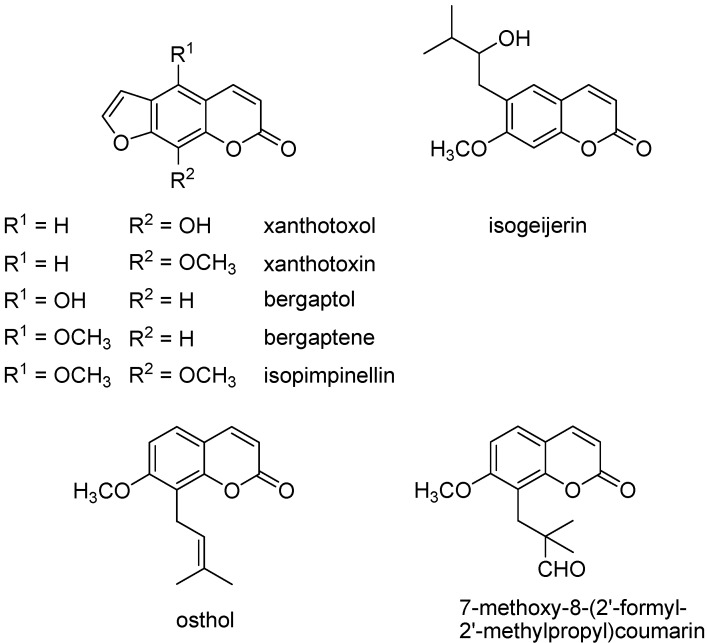
Structures of coumarins occurring in hexane extract of* M. tomentosa* flowers.

The GC-MS analysis (Table 1) identified 27 compounds, representing the 90.1% of the extract, that is composed in 77.9% by coumarin derivatives (Figure 1), a heterogeneous group of compounds with very different pharmacological activities, occurring in more than 700 species of the Apiaceae family [4]. More than half of the identified compounds (62.9%) belonged to the furanocoumarins class, a very interesting group of compounds well known for their capability to inhibit the germination of seeds behaving as inhibitors or as exogenous allelopathic agents [5]. Furanocoumarins are phototoxic and photogenotoxic natural constituents occurring in a broad variety of plants used in cosmetics, food and drugs but especially known for their photosensitizing UV-induced action and used in many drugs for the photodynamic destruction of tumor cells and other skin disorders such as psoriasis and vitiligo [6]. Grapefruit juice is considered as the most important dietary source of furanocoumarins [7]. The abundance of this class of compounds in this extract could well explain the skin-staining property.

**Table 1:** GC-MS analysis of *M. tomentosa* petroleum ether extract.

R_t_^ a^	Component	Id. ^b^	1% ^c^	2% ^c^
63.62	(*Z*)-9-Hexadecenoic acid methyl ester; methyl palmitoleate	1, 2, 3		0.1
64.54	Hexadecanoic acid methyl ester; methyl palmitate	1, 2, 3		1.6
68.61	5-Demethoxyisoimpinellin; xanthotoxin	2	23.6	22.9
69.57	Bergaptene	2	3.6	3.4
70.79	(*Z.Z*)-9,12-Octadecadienoic acid methyl ester; methyl linoleate	1, 2, 3		0.4
71.02	(*Z.Z.Z*)-9,12,15-Octadecatrienoic acid methyl ester; methyl linolenate	1, 2, 3		0.3
72.05	Octadecanoic acid methyl ester; methyl stearate	1, 2, 3		0.1
72.42	7-Methoxy-8-isopentenylcoumarin; osthol	2	6.4	5.6
73.34	(6*E*.10*E*)-7,11,15-Trimethyl-3-methylene-hexadeca-1,6,10,14-tetraene; β-springene	2	0.9	1
75.74	Isopimpinellin	2	17.8	18.8
76.39	7-Methoxy-8-(2'-formyl-2'-methylpropyl)coumarin	2	7.7	7.9
79.6	Xanthotoxol	2	17.9	17.4
80.74	Bergaptol	2	t	0.2
74.91	7-Methoxy-6-(2'-oxo-3'-methylbutyl)coumarin; isogeijerin	2	0.9	0.5
84.33	Pentacosane	1, 2, 3	0.5	0.6
85.34	Docosanoic acid methyl ester; methyl behenate	1, 2, 3		0.2
90.27	Heptacosane	1, 2, 3	1.2	1.4
91.16	Tetracosanoic acid methyl ester; methyl lignocerate	1, 2, 3		0.3
93.05	Octacosane	1, 2, 3	0.2	0.2
95.93	Nonacosane	1, 2, 3	7.2	7.6
96.69	Hexacosanoic acid methyl ester; methyl cerotate	1, 2		0.2
98.41	Triacontane	1, 2, 3	0.2	0.2
101.03	Hentriacontane	1, 2	1.8	1.9
101.85	Octacosanoic acid methyl ester	1, 2		0.1
107.73	Triacontanoic acid methyl ester; methyl melissate	2		0.1
112.67	Hop-22(29)-en-3β-ol	2	0.2	0.2
	**Total**		**90.1**	**93.2**

^a^: retention times on the HP 5MS column; ^b^, Identification: 1: retention time identical to authentic compound, 2: comparison of mass spectra with MS libraries and spectral data obtained from Wiley Subscription Services, Inc. (Hoboken, NJ, USA), 3 = co-injection with authentic compound; ^c^: **1**: *Magydaris* flowers extracted with petroleum ether; **2**: *Magydaris* **1** + CH_2_N_2_.

In addition, 12% of the extract has been shown to be mainly constituted by saturated hydrocarbons with an odd number of carbon atoms in the chain,* i.e*., nonacosane (7.2%) and hentriacontane (1.8%) that are universally distributed as cuticular waxes on the plant surfaces.

The GC-MS analysis of the extract, treated with diazomethane, confirmed the above data and showed the presence of a small amount (3.4%) of long chain fatty acids (from C-16 to C-30) detected in the form of methyl esters.

It is worth pointing out the differences in composition in the present study on *M. tomentosa* flowers from the previously investigation [2] of the extract from the same source. Polarity of solvent, status of plant material and analysis method can explain these differences. In fact, only osthol is a common metabolite. The more polar coumarins (having more than one hydroxyl group) were detected in the polar solvent (acetone) extract of powdered flowers by preparative column chromatography whereas the infusion in apolar solvent (petroleum ether) of the intact flowers released less polar coumarins identified by more sensitive GC-MS analysis.

The most abundant compound is the xanthotoxin (23.6%), a drug used to treat psoriasis, eczema, vitiligo and some cutaneous lymphomas in conjunction with exposing the skin to UVA light from lamps or sunlight. Xanthotoxin has been previously reported to possess a significant and good antiproliferative activity (10 µg/mL) against MCF-7 cell line [8] and it has been also tested against MK-1, HeLa and B16F10 cell lines [9].

Other abundant compounds are xanthotoxol and isopimpinellin present in the same concentration (17.9% and 17.8%, respectively). Xanthotoxol has also been evaluated for its cytotoxicity against MCF-7, showing an IC_50_ value similar to xanthotoxin (11.92 µg/mL) [10]. Isompimpinellin, joining the structural characteristics of xanthotoxin and bergaptene in one molecule, has been reported as a P450 inhibitor and blocking agent of DNA adduct formation with carcinogenic polycyclic aromatic hydrocarbons in the human breast MCF-7 adenocarcinoma cell line [11]. A relevant amount (7.7%) of a rare coumarin, 7-methoxy-8-(2'-formyl-2'-methylpropyl)-coumarin has been detected in the extract. Its occurrence has been reported in grapefruit peel essential oil [12], in the *Seseli tortuosum* (Apiaceae) [13] and in *Murraya paniculata* (Rutaceae) [14]. No biological activity of this compound was described up to now.

It’s also interesting the presence in the extract of the osthole (6.4%) that it has just been showed to possess antiproliferative capability with an IC_50_ value of 25.8 µM (6.30 µg/mL) against MCF-7 cell line [15]; furthermore, osthole and bergapten (3.6%) are known for the ability of the former to induce apoptosis on target cells [16], whereas the latter one has been described as a potent topoisomerase I inhibitor [17].

On these bases, we decided to test the *M. tomentosa* flowers petroleum ether extract for its antiproliferative activity against three cancer cell lines (Table 2), including murine monocyte/macrophages, J774A1, human melanoma cells, A375, and human breast cancer, MCF-7 and their antiproliferative activity was determined through the MTT conversion assay [18].

After 72 h of incubation with diluted solutions of the extract, the A375 cells showed no changes in cell viability, whereas J774A.1 cells were slightly affected. Good results have been obtained with MCF-7 cell line, which showed an IC_50_ of 0.94 µg/mL better than the single compounds constituting the most part of the extract. This finding could be explained by some synergic effects arising among the furanocoumarins occurring in the extract.

**Table 2:** *In vitro* anti-proliferative activity of *M. tomentosa* flowers petroleum ether extract against J774.A1 macrophages, MCF-7 human breast cancer and A375 human melanoma cells after 72 h of exposure.

	IC_50_ at 72 h		
	J774A.1	MCF7	A375
*M. tomentosa* flowers petroleum ether extract	71.52 ± 3.44	0.94 ± 0.6	nd
6-Mercaptopurine	0.003	48.23	142.36

IC_50_ values for different cancer cell lines are expressed in µg/mL for extract and in µM for 6-mercaptopurin, used as reference drug. The IC_50_ value is the concentration of extract or compound that affords 50% reduction in cell growth after 72 h of incubation. Values are expressed as mean ± SD, *n* = 3. nd, not detected.

## 3. Experimental Section

### 3.1. Plant Material

The umbels of *Magydaris tomentosa* (Desf.) W. D. J. Koch ex DC, were collected from blooming plants in Sicily, at Piana degli Albanesi (Palermo), in spring of 2013. Samples of the studied material (specimen number: PAL 13/47MB), identified by Emanuele Schimmenti, are kept in the Department STEBICEF, University of Palermo, Palermo, Italy.

### 3.2. Extraction Procedure

Fresh plant material (120 g) was extracted with petroleum ether (300 mL) at room temperature for two hours, to give, after the evaporation of the solvent, 2.1 g of extract. A portion of the extract (100 mg) was successively treated with a solution of CH_2_N_2_ in diethyl ether.

### 3.3. Reagents

Unless stated otherwise, all reagents and compounds were obtained from Sigma Chemicals Company (Sigma, Milan, Italy).

### 3.4. GC-MS Analysis: Qualitative and Quantitative Analyses of the Extract

The samples were analyzed to determine the chemical components at the “Department of Pharmacy” of the University of Naples “Federico II” by GC-MS. The GC-MS analyses were carried out with an Agilent 6850 Ser. II apparatus (Agilent Technologies, Santa Clara, CA, USA) coupled to an Agilent Mass Selective Detector MSD 5973 as previously described [19]. Identification of constituents was made as elsewhere reported [20].

### 3.5. Cell Lines Culture

J774A.1, cultured murine monocyte macrophages, A375 human melanoma cell line and MCF-7 human breast cancer cell line were purchased from American Tissue Culture Collection (ATCC, Rockville, MD, USA) and used to evaluate the antiproliferative activity of petroleum ether extract from* Magydaris tomentosa* flowers*.* All the media and sera were purchased from Hy-Clone (Euroclone, Paignton, Devon, UK); MTT [3(4,5-dimethylthiazol-2-yl)-2,5-phenyl-2H tetrazolium bromide] and 6-mercaptopurine (6-MP) were purchased from Sigma Chemicals. Cultured cells were grown in adhesion on Petri dishes and maintained with Dulbecco’s modified Eagle’s medium (DMEM) supplemented with 10% foetal calf serum (FCS), 25 mM HEPES, 2 mM glutamine, 100 U/mL penicillin and 100 µg/mL streptomycin at 37 °C in a Hera Cell humidified CO_2_ incubator (Kendro Laboratory, Hanau, Germany) with 5% CO_2_.

### 3.6. MTT Anti-Proliferative Assay

Cells (J774.A1, A375 and MCF7, 3.4 × 10^4^/well) were placed on 96-well microtiter plates and allowed to adhere for 2 h at 37 °C in 5% CO_2_ and 95% air. Thereafter, the medium was replaced with 90 µL of fresh medium and a 10 µL aliquot of serial dilutions of *Magydaris* petroleum ether extract (10–100 µg/mL), dissolved in DMSO and then diluted in DMEM, was added. Cells were incubated for 72 h. Cell viability was assessed through MTT assay [21]. Briefly, 25 µL of MTT (5 mg/mL) were added and the cells were incubated for additional 3 h. Thereafter, cells were lysed and the dark blue crystals solubilised with 100 µL of a solution containing 50% (v:v) N,N-dimethylformamide, 20% (w:v) SDS with an adjusted pH of 4.5. In our experiments, serial dilutions of 6-MP, as reference anti-proliferative drug, has been used. The cell viability was assessed through an MTT conversion assay [18]. The optical density (OD) of each well was measured with a microplate spectrophotometer (Titertek Multiskan MCC/340, Titertek Instruments Inc., Huntsville, AL, USA) equipped with a 620 nm filter. The test concentration which inhibits 50% of each cell population (IC50) was obtained by Probit Analysis (SPSS Version 12.0.1, Chicago, IL, USA). All the esperiments were carried out in triplicates. The viability of each cell line in response to treatment with graded concentrations of *Magydaris* petroleum ether extract, was calculated as:

% dead cells = 100 − (OD treated/OD control) × 100
(1)

## 4. Conclusions

Less polar coumarins occurring in flowers of *M. tomentosa* can be efficiently extracted with simple infusion of the intact blossoming tops in petroleum ether. This extract is mainly constituted of furanocoumarins with high biological activities. Comparing the literature data, the cytotoxicity of the extract resulted more powerful against MCF-7 cell line than single compounds tested until now. This fact can be explained by synergic effects among compounds constituting the extract.
